# Dismantling the tumoral cloak of self-protection

**DOI:** 10.1371/journal.pbio.3002104

**Published:** 2023-05-04

**Authors:** Mara B. Willis, Katherine Y. King

**Affiliations:** Department of Pediatrics, Division of Infectious Diseases, Texas Children’s Hospital and Baylor College of Medicine, Houston, Texas, United States of America

## Abstract

The mechanisms by which tumors promote hematopoiesis outside the bone marrow have been the subject of intense interest. This Primer explores a new PLOS Biology study that provides new insight into the mechanisms underlying this process, and may hold the key to disrupting generation of the immunosuppressive tumor microenvironment.

## Teaser

Tumors protect themselves from immune clearance by promoting extramedullary hematopoiesis. A new study in *PLOS Biology* provides insights into the mechanisms underlying this process, which may hold the key to disrupting generation of the immunosuppressive tumor microenvironment.

Tumors have ingenious means of self-protection. They can lure hematopoietic stem and progenitor cells (HSPCs), the body’s blood producing cells, away from their natural home in the bone marrow to new places, such as the spleen [1]. There, the HSPCs are instructed to make immunosuppressive myeloid cells that blunt antitumor immunity, thereby shielding the tumor in a cloak of self-protection.

The evidence for protective myelopoiesis in the setting of solid organ malignancies extends across multiple tumor types including breast, colon, and lung [2]. In patients with malignancies, a high neutrophil to lymphocyte ratio, a measure of the degree of myelopoiesis, portends a poor prognosis [3]. This is thought to be because production of myeloid cells, including myeloid-derived suppressor cells, inhibits cytotoxic immune responses by T cells and NK cells that are critical for antitumor immunity. Such immunosuppressive tumor microenvironments are a major obstacle in immunotherapeutic approaches to cancer. Thus, the mechanisms by which tumors raise this cloak of protection are the subject of intense interest.

**Fig 1:**
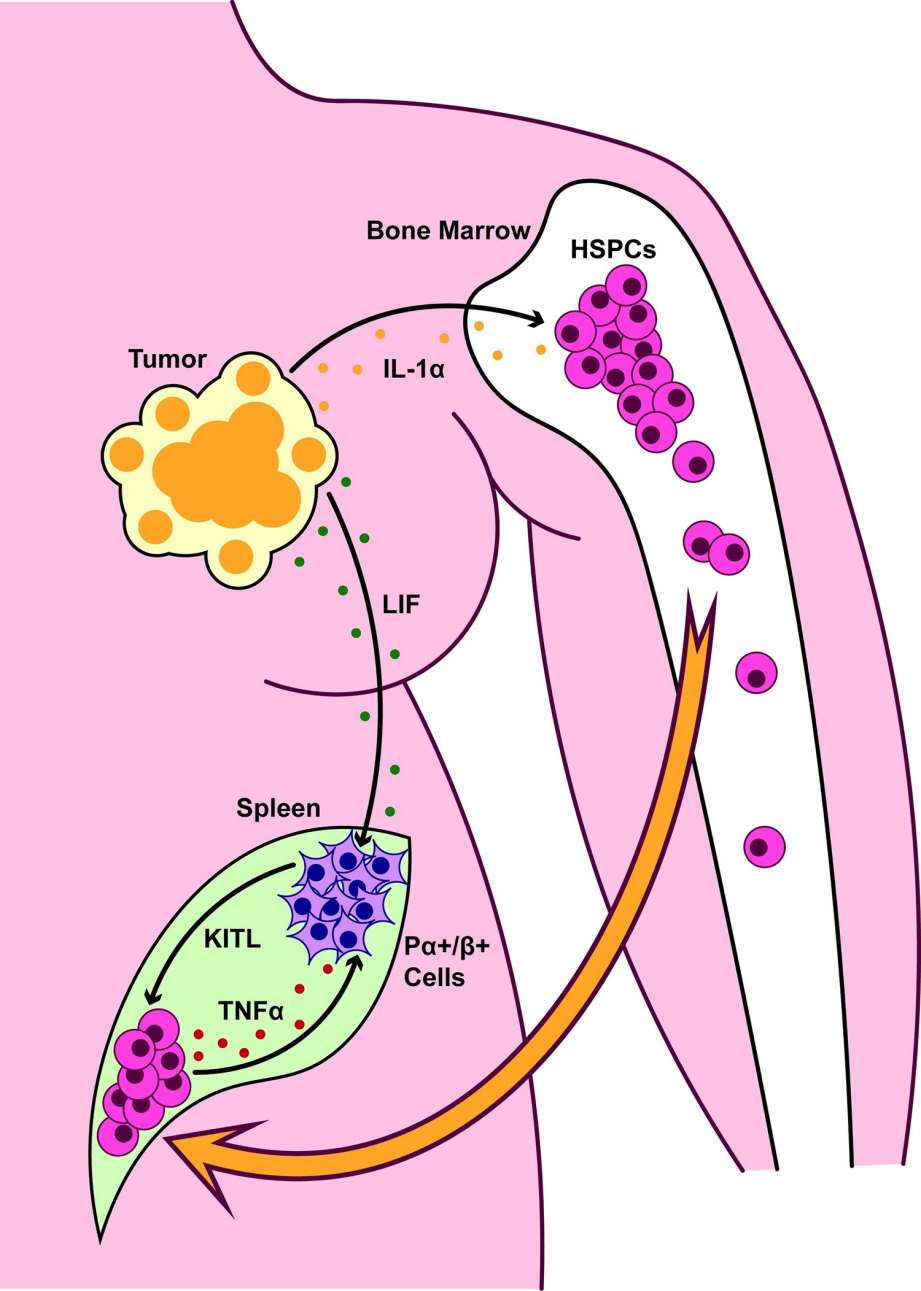
IL-1α and LIF produced by tumors promotes extramedullary hematopoiesis by inducing HSCP-derived TNFα and activation of splenic ABS cells. Tumor cells produce IL-1α, which activates TNFα expression by HSPCs. TNFα and tumor-derived LIF are sensed by splenic Pdgfra^+^/Pdgrfb^+^ stromal (ABS) cells, which produce cytokines such as CXCL12 and KITL (also known as SCF) to promote homing of HSPCs. Thus, tumor-derived factors promote splenic extramedullary hematopoiesis, a source of immunosuppressive myeloid cells that facilitate immune evasion. HSPC, hematopoietic stem and progenitor cell; LIF, leukemia inhibitory factor.

In a new article in *PLOS Biology*, Barisas and colleagues report a rigorous study that sought to understand how tumors attract and support extramedullary hematopoiesis (EMH) in the spleen [4] (Fig 1). They identify two complementary mechanisms by which tumors draw HSPCs to the spleen to produce myeloid cells, including neutrophils. Using a heterotopic tumor transplantation model, the investigators examined mechanisms of neutrophilia in the setting of breast cancer. They found that tumors produced the inflammatory cytokine IL-1α, which was found at increased levels in the circulation of tumor-bearing mice. Single-cell RNAseq revealed a pro-inflammatory gene signature in HSPCs at extramedullary sites in tumor-bearing mice, including expression of TNFα and NFκB, a common stress response regulator triggered by pro-inflammatory cytokines such as interferons and pathogen recognition receptors such as Toll-like receptors. Injection of IL-1α into mice was sufficient to induce TNFα production by HSPCs, suggesting a mechanism of direct communication between tumors and HSPCs.

Pro-inflammatory cytokines are known to have powerful effects on HSPCs, inducing their migration, division, and myeloid differentiation [5,6]. Indeed, a recent study showed that pro-inflammatory cytokines produced during murine sepsis were sufficient to induce myeloid differentiation and, more specifically, production of myeloid-derived suppressor cells (MDSCs) [7]. Cytokine-dependent production of MDSCs by HSPCs may be a natural mechanism by which the body down-regulates inflammatory responses to an acute infectious challenge.

In the study by Barisas and colleagues, deletion of *IL1A* from the tumor cells decreased TNFα and total splenic myeloid progenitors [4]. A population of Pdgfra^+^/Pdgrfb^+^ stromal (ABS) cells in the spleen strongly express TNFα receptor and the HSPC growth cytokine KITL (also known as SCF). In vitro studies indicated that these cells respond to TNFα to attract and support the growth of bone marrow-derived HSPCs in the spleen. Notably, tumor-bearing mice receiving IL-1 receptor blocking antibody had decreased neutrophilia.

Next, the investigators also demonstrated production of leukemia inhibitory factor (LIF) by tumor cells, which induced expansion of HSPC and myeloid progenitors in the spleen and promoted neutrophilia. LIF is a cytokine that inhibits differentiation, thereby promoting expansion of undifferentiated cells. LIF receptor was required for ABS cells to support hematopoiesis, including by the production of stem cell homing and growth factors such as CXCL12 and SCF. Thus, the tumor-derived IL-1 and LIF cooperate to promote EMH in cancer. The authors have yet to demonstrate that the myeloid cells resulting from tumor-derived IL-1 and LIF truly function as MDSCs. Nevertheless, the combined release of these two cytokines by tumors may represent an example of tumors coopting a natural homeostatic response to generate self-protective antitumor responses. Importantly, tackling this mechanism may be an effective avenue to overcome the immunosuppressive microenvironment that continues to represent a major barrier to immunotherapy in solid cancers [8].

Both the IL-1α receptor blocker anakinra and the IL-1β receptor blocker canakinumab have been the subject of clinical trials and intense interest in the cancer field following news that the CANTOS trial unexpectedly showed that use of canakinumab to block IL-1 signaling was associated with a lower incidence and mortality from lung cancer [9,10]. Unfortunately, two key follow up studies, CANOPY I and CANOPY II, have failed to meet primary endpoints, and studies using anakinra, canakinumab, or newer IL-1 inhibitors remain ongoing. The study by Barisas snd colleagues [4] may provide the additional insight needed to truly address immunosuppressive myeloid responses in cancer. In other words, blocking both IL-1 and LIF together may be much more effective than either alone. Future studies will determine whether antagonizing LIF or IL-1 or both is sufficient to reduce tumor progression either in the murine model or in humans.

## Abbreviations

EMH: extramedullary hematopoiesis
HSPC: hematopoietic stem and progenitor cell
LIF: leukemia inhibitory factor
MDSC: myeloid-derived suppressor cell

## References

[1] Wu C, Ning H, Liu M, Lin J, Luo S, Zhu W, et al. Spleen mediates a distinct hematopoietic progenitor response supporting tumor-promoting myelopoiesis. J Clin Invest. 2018;128(8):3425–3438. doi: 10.1172/JCI97973 29771686 PMC6063469

[2] Nakamura K, Smyth MJ. Myeloid immunosuppression and immune checkpoints in the tumor microenvironment. Cell Mol Immunol. 2020;17(1):1–12. doi: 10.1038/s41423-019-0306-1 31611651 PMC6952382

[3] Corbeau I, Jacot W, Guiu S. Neutrophil to Lymphocyte Ratio as Prognostic and Predictive Factor in Breast Cancer Patients: A Systematic Review. Cancers (Basel). 2020;12(4):958. 32295078 10.3390/cancers12040958 PMC7226461

[4] Barisas DAG, Ul Kabir A, Wu J, Krchma K, Kim M, Subramanian M, et al. Interleukin-1α and Leukemia Inhibitory Factor Promote Extramedullary Hematopoiesis. PLoS Biol. 2023; 21(5):e3001746. doi: 10.1371/journal.pbio.3001746 37134077 PMC10155962

[5] Hormaechea-Agulla D, Le DT, King KY. Common Sources of Inflammation and Their Impact on Hematopoietic Stem Cell Biology. Curr Stem Cell Rep. 2020;6(3):96–107. doi: 10.1007/s40778-020-00177-z 32837857 PMC7429415

[6] Pietras EM, Mirantes-Barbeito C, Fong S, Loeffler D, Kovtonyuk LV, Zhang S, et al. Chronic interleukin-1 exposure drives haematopoietic stem cells towards precocious myeloid differentiation at the expense of self-renewal. Nat Cell Biol. 2016;18(6):607–618. doi: 10.1038/ncb3346 27111842 PMC4884136

[7] Morales-Mantilla DE, Kain B, Le D, Flores AR, Paust S, King KY. Hematopoietic stem and progenitor cells improve survival from sepsis by boosting immunomodulatory cells. elife. 2022;11:e74561. doi: 10.7554/eLife.74561 35166205 PMC8846591

[8] Lythgoe MP, Prasad V. Repositioning canakinumab for non-small cell lung cancer-important lessons for drug repurposing in oncology. Br J Cancer. 2022;127(5):785–787. doi: 10.1038/s41416-022-01893-5 35739301 PMC9427732

[9] Zhang J, Veeramachaneni N. Targeting interleukin-1beta and inflammation in lung cancer. Biomark Res. 2022;10(1):5. 35086565 10.1186/s40364-021-00341-5 PMC8796434

[10] Ridker PM, MacFadyen JG, Thuren T, Everett BM, Libby P, Glynn RJ, et al. Effect of interleukin-1beta inhibition with canakinumab on incident lung cancer in patients with atherosclerosis: exploratory results from a randomised, double-blind, placebo-controlled trial. Lancet. 2017;390(10105):1833–1842. 28855077 10.1016/S0140-6736(17)32247-X

